# A comprehensive analysis of antimicrobial resistance of clinical *emm*89 *Streptococcus pyogenes* in Japan

**DOI:** 10.1093/jacamr/dlaf017

**Published:** 2025-02-19

**Authors:** Weichen Gong, Masayuki Ono, Masaya Yamaguchi, Daisuke Motooka, Yujiro Hirose, Kotaro Higashi, Momoko Kobayashi, Eri Ikeda, Tomoko Sumitomo, Rumi Okuno, Takahiro Yamaguchi, Ryuji Kawahara, Hitoshi Otsuka, Noriko Nakanishi, Yu Kazawa, Chikara Nakagawa, Ryo Yamaguchi, Hiroo Sakai, Yuko Matsumoto, Tadayoshi Ikebe, Shigetada Kawabata

**Affiliations:** Department of Microbiology, Osaka University Graduate School of Dentistry, Osaka, Japan; Graduate School of Medicine, Osaka University, Osaka, Japan; Department of Microbiology, Osaka University Graduate School of Dentistry, Osaka, Japan; Bioinformatics Research Unit, Osaka University Graduate School of Dentistry, Osaka, Japan; Department of Microbiology, Osaka University Graduate School of Dentistry, Osaka, Japan; Bioinformatics Research Unit, Osaka University Graduate School of Dentistry, Osaka, Japan; Laboratory of Microbial Informatics, Microbial Research Center for Health and Medicine, National Institutes of Biomedical Innovation, Health and Nutrition, Osaka, Japan; Bioinformatics Center, Research Institute for Microbial Diseases, Osaka University, Osaka, Japan; Center for Infectious Diseases Education and Research, Osaka University, Osaka, Japan; Bioinformatics Center, Research Institute for Microbial Diseases, Osaka University, Osaka, Japan; NGS Core Facility, Research Institute for Microbial Diseases, Osaka University, Osaka, Japan; Integrated Frontier Research for Medical Science Division, Institute for Open and Transdisciplinary Research Initiatives (OTRI), Osaka University, Osaka, Japan; Department of Microbiology, Osaka University Graduate School of Dentistry, Osaka, Japan; Department of Microbiology, Osaka University Graduate School of Dentistry, Osaka, Japan; Department of Microbiology, Osaka University Graduate School of Dentistry, Osaka, Japan; Laboratory of Microbial Informatics, Microbial Research Center for Health and Medicine, National Institutes of Biomedical Innovation, Health and Nutrition, Osaka, Japan; Department of Microbiology, Osaka University Graduate School of Dentistry, Osaka, Japan; Department of Microbiology, Osaka University Graduate School of Dentistry, Osaka, Japan; Department of Oral Microbiology, Graduate School of Biomedical Sciences, Tokushima University, Tokushima, Japan; Department of Microbiology, Tokyo Metropolitan Institute of Public Health, Tokyo, Japan; Department of Bacteriology, Osaka Institute for Public Health, Osaka, Japan; Department of Bacteriology, Osaka Institute for Public Health, Osaka, Japan; Department of Public Health Sciences, Yamaguchi Prefectural Institute for Public Health and Environment, Yamaguchi, Japan; Department of Infectious Diseases, Kobe Institute of Health, Hyogo, Japan; Department of Microbiology, Fukushima Prefectural Institute for Public Health, Fukushima, Japan; Division of Microbiology, Kyoto City Institute of Health and Environmental Sciences, Kyoto, Japan; Sapporo Public Health Office, Hokkaido, Japan; Niigata City Institute of Public Health and the Environment, Niigata, Japan; Microbiological Testing and Research Division, Yokohama City Institute of Public Health, Kanagawa, Yokohama, Japan; Department of Bacteriology I, National Institute of Infectious Diseases, Tokyo, Japan; Department of Microbiology, Osaka University Graduate School of Dentistry, Osaka, Japan; Center for Infectious Diseases Education and Research, Osaka University, Osaka, Japan

## Abstract

**Objectives:**

*Streptococcus pyogenes* is involved in a wide range of diseases, including pharyngitis and life-threatening invasive infections. Increasing prevalence of antimicrobial resistance (AMR) has been reported worldwide in various bacteria, limiting the use of antibiotics in infection cases. The present study investigated the AMR of most prevalent *S. pyogenes emm* types, including *emm*89 strains in Japan.

**Methods:**

A total of 368 previously identified *S. pyogenes* isolates (311 *emm*89 strains and 57 of other *emm* types), which were previously isolated from patients with invasive and non-invasive infections throughout Japan, were used in the analyses. The minimum inhibitory concentrations of seven antibiotics, including penicillin-G, azithromycin (AZM) and clindamycin, were determined, and whole-genome sequences of AMR-associated genes were screened.

**Results:**

We identified 47 resistant strains, of which 91.49% (43/47) were resistant to AZM and/or clindamycin. A strong correlation was observed between non-invasive phenotypes and AMR. Whole-genome analysis indicated the wide distribution of three AMR-related genes, *ermT*, *folP* and *lmrP*, among the *emm*89 strains. Additionally, *tetO* was detected in tetracycline-resistance and *soxS* and *mel* was detected in chloramphenicol-resistance only in *emm*4 strains.

**Conclusions:**

The high prevalence of *S. pyogenes* resistance to AZM and/or clindamycin poses a threat to public health in Japan; thus, the development of next-generation antimicrobial therapies is imperative.

## Introduction

The Gram-positive bacterium, *Streptococcus pyogenes*, also known as Group A *Streptococcus*, causes a wide range of infectious diseases, including pharyngitis, tonsillitis, scarlet fever and life-threatening, severe, invasive infections. In severe, invasive *S. pyogenes* infections, the most severe manifestation is streptococcal toxic shock syndrome (STSS), which is characterized by sudden onset of shock, multi-organ failure and high mortality.^1^ The first case of STSS caused by *S. pyogenes* in Japan was reported in 1992, and the number of cases has continued to increase, especially in recent years.^2,3^ For the treatment of patients with STSS, the combination therapy of multiple antibiotics, including β-lactam antibiotics, macrolides and lincosamides, has been proven to substantially reduce both mortality and morbidity.^4^ Although β-lactam antibiotics are effective in both non-invasive and invasive *S. pyogenes* infections, azithromycin (AZM) or clindamycin is administered to patients with penicillin allergy. Although *S. pyogenes* remains universally sensitive to β-lactam, macrolide- and lincosamide-resistance frequently result in recurrent infection, treatment failure and poor patient outcomes.^5^ Genotyping of the *emm* gene encoding the cell-surface M protein is usually applied to clinical *S. pyogenes* isolates obtained from patients.^6^ The predominant *emm* types found in STSS are 1, 89, 12 and 3 (arranged in descending order of prevalence), and account for more than 70% of the STSS isolates in Japan. Approximately 71% of macrolide- and lincosamide-resistant *emm*1 strains carry *mefA/E* or *ermB* genes, but the antimicrobial genes important for resistance in *emm*89 strains remain unclear.^3^

In the present study, we examined antimicrobial resistance (AMR) and AMR-associated genes in 311 *emm*89 isolates and another 57 isolates from other *emm* types, which were previously obtained from patients throughout Japan with invasive and non-invasive *S. pyogenes* infections. By understanding the AMR of *emm*89 *S. pyogenes* and its AMR-associated genes, health care practitioners can administer targeted medication for patients with AMR *S. pyogenes* infection.

## Patients and methods

### Bacterial strains

A total of 311 *emm*89 and 57 other *emm*-type *S. pyogenes* strains were collected from 13 prefectures in Japan from 2011 to 2021; the whole-genome sequencing was performed in previous studies.^7,8^ Clinical isolates were collected from public health institutions in Japan. We defined the strains collected as STSS according to the Infectious Diseases Control Law in Japan. Non-invasive strains were defined based on diagnostic names, including asymptomatic, pharyngitis, tonsillitis or non-invasive infections.^8^ Of all the isolates, 311 were typed as *emm*89 strains and the other isolates were typed as *emm*4 (n = 24), *emm*12 (n = 19), *emm*3.95 (n = 7), *emm*28.10 (n = 1), *emm*48.1 (n = 1), *emm*11 (n = 4) and *emm*81.0 (n = 1) (Figure S1, available as Supplementary data at *JAC-AMR* Online).^8^

### Minimum inhibitory concentration assay

The minimum inhibitory concentration (MIC) assay was performed in accordance with the Clinical Laboratory Standards Institute (CLSI) guidelines as previously described, with a minor modification.^9,10^ Regarding the modification, we performed anaerobic culture using AnaeroPack (Mitsubishi GAS Chemical Co. Inc., Tokyo, Japan) in the presence of antimicrobial agents in order to reliably culture clinical isolates. Initially, *S. pyogenes* strains were cultured at 37°C in an atmosphere containing 5% CO_2_ in brain heart infusion (BHI) broth (BD Bacto, Maryland, USA). For the MIC assay, an overnight bacterial culture was diluted 1:30 with fresh BHI and grown to the logarithmic phase (optical density at 600 nm, OD_600_ = 0.4–0.5). The bacterial culture was then washed and diluted 1:100 with BHI broth. Then, 5 µL of bacteria were added to the individual wells of a 96-well plate containing 175 µL Mueller–Hinton broth supplemented with 5% lysed horse blood (Kyokuto Pharmaceutical Industrial Co., Ltd, Tokyo, Japan) and 20 µL of 2-fold serial dilutions of the following antimicrobials: penicillin-G (PG; 0.125–8 mg/L ), AZM (0.031–2 mg/L), erythromycin (ERY; 0.016–1 mg/L), tetracycline (TC; 0.125–8 mg/L), chloramphenicol (CHL; 0.25–16 mg/L), levofloxacin (LFX; 0.125–8 mg/L) or clindamycin (CLI; 0.016–1 mg/L). PG, AZM, TC, CHL, LFX and CLI were purchased from Tokyo Chemical Industry Co., Ltd (Tokyo, Japan). ERY was purchased from Sigma-Aldrich Inc. (St. Louis, MO, USA) (Table S1). Bacterial growth after 24 h at 37°C was spectrophotometrically measured at 600 nm using a spectrophotometer plate reader (Tecan Infinite F Plex; Tecan Group Ltd, Zurich, Switzerland). OD_600_ values of <0.06 were considered to represent the complete inhibition of bacterial growth.

### Sequence-based profiling of AMR-related genes

Sequence-based profiling was performed as previously described.^10^ Briefly, to detect AMR-related genes, sequence-based profiling was performed using ARIBA v.2.14.4 referring to the comprehensive antibiotic resistance database (CARD) v.3.2.7.^11,12^ The minimum percentage identity of the assemblies was set at 90. The analysed data were visualized using Phandango software.^11^ For genes that were determined as ‘interrupted’ or ‘partial’ sequences in each genome, the presence of complete sequences was examined by searching assembled genomes using BLAST v.2.13.0. For strains for which the detected genes did not explain their resistance, sequence-based profiling using MEGARes and AMR++ v.3.0 was performed.^13^

### AMR-related gene detection using genome-wide association studies

All the processes and analyses were performed using the National Institute of Genetics (NIG) supercomputer and supercomputer for quest to unsolved interdisciplinary datascience (SQUID) at D3 Center of Osaka University (Osaka, Japan). Genome-wide association studies (GWASs) were performed using the *emm*89 strains following the previously constructed workflow for bacterial GWASs.^8^ Briefly, pan-genome sequences were calculated and a core gene alignment and distribution of all genes among the strains were generated using Roary v.3.12.0.^14^ Single-nucleotide polymorphisms and indels (SNPs/indels) were extracted from core genes conserved among more than 99% of the strains using snp-sites v.2.5.1 and bcftools v.1.9. GWASs were performed using PySeer v.1.3.4 to investigate the associations between phenotypes and genotypes, including SNPs/indels and genes.^15^ The VCF file of the SNPs/indels or the gene distribution matrix was designated as the genotype. Phenotypes were defined according to the resistance or susceptibility to each antibiotic.

### Data visualization

Hiplot (ORG) (https://hiplot.org), a comprehensive and easy-to-use web service for boosting publication-ready biomedical data visualization, was used to visualize the following data: (i) the correlation between AMR and invasive streptococcal infection was visualized using a stacked bar chart and (ii) AMR patterns and their relationships were visualized using a Venn diagram.^16^

Using the ggplot2 package in R, the results of the GWASs were visualized as a Manhattan plot (for the SNP/indel-based GWAS) and a volcano plot (for the gene-based GWAS). Heatmaps of strains possessing significant variants were generated using the gplots package in R.

### Statistical analysis

The correlation between AMR and invasiveness was statistically analysed using the chi-square test in Excel (v. 16.81; Microsoft, Redmond, WA, USA). Differences were considered statistically significant at *P *< 0.05. The significance levels of GWASs were determined using a permutation test that applies the five-percentile value of the minimal *P* values in 1000 times-iterated calculations with randomized phenotypes. Significant variants were further curated to exclude false positives due to errors in *de novo* assembly.

## Results

### Correlations between AMR and invasiveness of emm89 S. pyogenes

Previously, 311 *emm*89 and 57 other *emm*-type *S. pyogenes* strains were collected from 13 prefectures in Japan from 2011 to 2021. Of all the isolates, 311 were typed as *emm*89 strains and the other isolates were typed as *emm*4 (n = 24), *emm*12 (n = 19), *emm*3.95 (n = 7), *emm*28.10 (n = 1), *emm*48.1 (n = 1), *emm*11 (n = 4) and *emm*81.0 (n = 1) (Figure S1).^8^ Antibiotic susceptibility and the correlation between AMR and the invasiveness of the isolates were analysed to elucidate the infection status of *emm*89 isolates in Japan.

**Figure 1.**
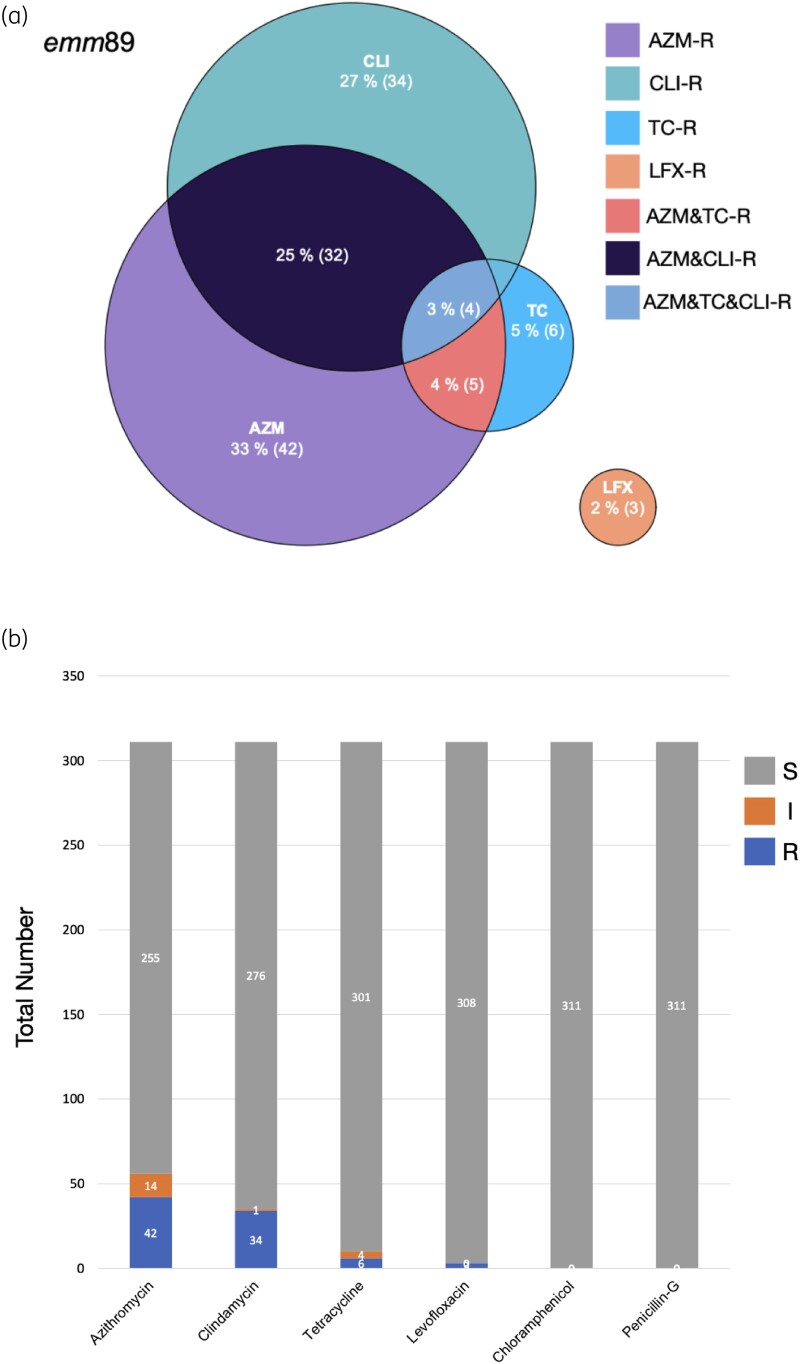
AMR of *S. pyogenes* throughout Japan. (a) Venn diagram showing seven grouped populations of AMR *emm*89 *S. pyogenes*. AZM-R, azithromycin-resistance; TC-R, tetracycline-resistance; CLI-R, clindamycin-resistance; LFX-R, levofloxacin-resistance; AZM&TC-R, azithromycin and tetracycline-resistance; AZM&CLI-R, azithromycin and clindamycin-resistance; AZM&TC&CLI-R, azithromycin, tetracycline and clindamycin-resistance. (b) Stacked barplot showing the AMR status of 311 isolates of *S. pyogenes emm*89 strains. Upper, middle and lower bars indicate ‘sensitive’ (S), ‘intermediate’ (I) and ‘resistant’ (R), respectively.

First, the MICs of seven antibiotics, PG, ERY, AZM, TC, levofloxacin, chloramphenicol and clindamycin, were measured against all 368 isolates (Figure 1b; Tables S1 and S2). Strains resistant to one or more antibiotics, except for PG, were found (Figure 1a and b). All AZM-resistant *emm*89 strains were also resistant to ERY (Figure S2; Table S2). Thus, for the following analyses, we used the result of AZM as a representative of macrolides because AZM is more frequently administered clinically.

AMR patterns were defined based on the combination of antibiotics that each strain could resist. The AMR patterns varied across different *emm* types, and seven patterns were identified in *emm*89 strains (Figure 1a and b). Among the *emm*89 isolates resistant to at least one antibiotic, 91.49% (43/47) of them were resistant to AZM and/or clindamycin, which includes resistance to AZM, clindamycin, AZM&CLI (concurrent resistance to AZM and clindamycin), AZM&TC (concurrent resistance to AZM and TC) or AZM&CLI&TC (concurrent resistance to AZM, clindamycin and TC) (Figure 1a and b; Table 1; Table S2).

**Table 1.** Detailed information of AMR in all 368 *S. pyogenes* clinical isolates

*emm* type	Strains	MIC (mg/L)						
		Penicillin-G	Azithromycin	Tetracycline	Chloramphenicol	Levofloxacin	Clindamycin	Erythromycin
emm89.0	**KB01**	<0.125	1(I)	<0.125	2	0.5	0.063	0.125
emm89.0	**KB02**	<0.125	1(I)	<0.125	1	1	0.063	0.125
emm89.0	**KB03**	<0.125	1(I)	<0.125	1	0.5	0.063	0.125
emm89.0	**KB04**	<0.125	1(I)	0.5	1	0.25	0.031	0.125
emm89.0	**KB05**	<0.125	0.5	0.125	1	0.5	0.063	0.063
emm89.0	**KB06**	<0.125	0.5	0.125	1	0.25	0.063	0.125
emm89.0	**KB07**	<0.125	0.5	<0.125	1	1	0.031	0.125
emm89.0	**KB08**	<0.125	≥ 2(R)	≥ 8(R)	1	0.25	0.031	≥ 1(R)
emm89.0	**KB09**	<0.125	0.125	0.125	1	0.5	0.031	0.25
emm89.0	**KB10**	<0.125	0.25	<0.125	1	0.5	0.016	0.25
emm89.0	**KB11**	<0.125	1(I)	<0.125	2	0.5	0.063	0.125
emm89.0	**KB12**	<0.125	1(I)	<0.125	2	0.5	0.031	0.125
emm89.0	**KB13**	<0.125	0.5	<0.125	1	1	0.063	0.125
emm89.0	**YG40**	<0.125	1(I)	<0.125	2	0.5	0.063	0.063
emm89.0	**YG41**	<0.125	≤0.031	2	1	0.5	0.031	0.125
emm89.0	**YG42**	<0.125	≤0.031	2	1	0.25	0.063	0.063
emm89.0	**YG43**	<0.125	≤0.031	2	<0.25	<0.125	0.063	0.125
emm89.0	**YG44**	<0.125	≤0.031	2	1	0.5	≥ 1(R)	0.125
emm89.0	**YG45**	<0.125	1(I)	≥ 8(R)	2	0.25	≥ 1(R)	0.5(I)
emm89.0	**YG46**	<0.125	≤0.031	4(I)	1	0.5	0.063	0.125
emm89.0	**OS50**	<0.125	≤0.031	2	1	0.5	0.063	0.125
emm89.0	**OS51**	<0.125	≤0.031	4(I)	1	0.5	0.031	0.125
emm89.0	**OS52**	<0.125	≤0.031	4(I)	1	0.25	0.063	0.25
emm89.0	**OS53**	<0.125	0.5	<0.125	1	0.5	0.063	0.25
emm89.0	**OS54**	<0.125	0.5	<0.125	1	0.25	0.031	0.125
emm89.0	**OS55**	<0.125	≥ 2(R)	≥ 8(R)	1	2	≥ 1(R)	≥ 1(R)
emm89.0	**OS56**	<0.125	0.25	<0.125	1	0.25	0.016	0.25
emm89.0	**OS57**	<0.125	0.5	<0.125	2	0.5	0.063	0.25
emm89.0	**OS58**	<0.125	≥ 2(R)	≥ 8(R)	1	0.5	≥ 1(R)	≥ 1(R)
emm89.0	**OS59**	<0.125	0.5	<0.125	2	0.5	0.063	0.063
emm89.0	**OS60**	<0.125	0.5	<0.125	1	0.5	0.063	0.25
emm89.0	**OS61**	<0.125	0.5	<0.125	1	1	0.063	0.125
emm89.0	**OS62**	<0.125	≥ 2(R)	<0.125	1	0.5	≥ 1(R)	≥ 1(R)
emm89.0	**OS63**	<0.125	0.5	<0.125	1	0.25	0.063	0.125
emm89.0	**OS64**	<0.125	0.5	<0.125	1	0.5	0.063	0.063
emm28.10	**F7401**	<0.125	≥ 2(R)	<0.125	2	0.25	≥ 1(R)	≥ 1(R)
emm4.0	**F7412**	<0.125	≥ 2(R)	<0.125	1	0.25	0.063	≥ 1(R)
emm4.0	**F7419**	<0.125	0.5	<0.125	1	0.25	0.063	0.063
emm89.0	**FS14**	<0.125	0.25	<0.125	1	0.5	0.016	0.125
emm4.0	**F7432**	<0.125	≥ 2(R)	<0.125	2	0.5	0.063	≥ 1(R)
emm4.0	**F7448**	<0.125	≥ 2(R)	<0.125	2	0.5	0.031	≥ 1(R)
emm89.0	**FS15**	<0.125	0.125	<0.125	0.25	0.5	0.031	0.063
emm12.0	**F7469**	<0.125	≥ 2(R)	≥ 8(R)	2	2	≥ 1(R)	≥ 1(R)
emm3.95	**F7477**	<0.125	0.25	<0.125	1	0.25	0.063	0.063
emm12.7	**F7478**	<0.125	1(I)	<0.125	2	0.25	0.063	0.125
emm12.0	**F7515**	<0.125	≥ 2(R)	≥ 8(R)	2	2	≥ 1(R)	≥ 1(R)
emm4.0	**F7516**	<0.125	0.25	<0.125	2	0.25	0.031	0.063
emm12.0	**F7517**	<0.125	≥ 2(R)	≥ 8(R)	2	1	≥ 1(R)	≥ 1(R)
emm3.95	**F7527**	<0.125	0.25	<0.125	0.5	0.125	<0.016	0.063
emm89.0	**FS16**	<0.125	0.5	<0.125	1	1	0.063	0.125
emm12.7	**F7529**	<0.125	0.5	<0.125	1	0.25	0.063	0.063
emm12.0	**F7544**	<0.125	0.5	<0.125	1	0.25	0.063	0.063
emm89.0	**FS17**	<0.125	0.25	<0.125	2	0.25	0.031	0.063
emm89.0	**FS18**	<0.125	0.5	<0.125	1	0.5	0.063	<0.016
emm12.0	**F7587**	<0.125	≥ 2(R)	≥ 8(R)	2	2	≥ 1(R)	≥ 1(R)
emm12.0	**F7600**	<0.125	0.5	<0.125	1	0.25	0.063	0.063
emm12.7	**F7601**	<0.125	≥ 2(R)	≥ 8(R)	4	0.25	≥ 1(R)	≥ 1(R)
emm4.0	**F7637**	<0.125	≥ 2(R)	0.5	0.5	0.25	0.031	≥ 1(R)
emm12.0	**F7638**	<0.125	0.5	<0.125	1	0.5	0.063	0.125
emm89.24	**FS19**	<0.125	0.5	<0.125	1	0.5	0.063	0.125
emm4.0	**F7730**	<0.125	0.5	<0.125	1	0.25	0.063	0.063
emm3.95	**F7733**	<0.125	0.25	<0.125	1	0.25	0.063	0.063
emm89.0	**FS20**	<0.125	1(I)	<0.125	2	0.25	0.063	0.063
emm89.0	**FS21**	<0.125	0.25	<0.125	1	0.5	<0.016	0.125
emm89.0	**FS22**	<0.125	0.5	<0.125	1	1	0.031	0.063
emm89.0	**FS23**	<0.125	0.25	<0.125	1	1	<0.016	0.125
emm12.0	**F7886**	<0.125	≥ 2(R)	≥ 8(R)	2	1	≥ 1(R)	≥ 1(R)
emm48.1	**F7920**	<0.125	≥ 2(R)	≥ 8(R)	2	0.25	≥ 1(R)	≥ 1(R)
emm89.0	**FS24**	<0.125	0.25	<0.125	0.5	0.25	0.016	0.125
emm89.0	**FS25**	<0.125	1(I)	<0.125	1	0.25	0.063	0.5(I)
emm12.0	**F7924**	<0.125	≥ 2(R)	≥ 8(R)	2	1	≥ 1(R)	≥ 1(R)
emm12.0	**F7936**	<0.125	≥ 2(R)	≥ 8(R)	2	2	≥ 1(R)	≥ 1(R)
emm89.0	**FS26**	<0.125	1(I)	<0.125	1	0.5	0.031	0.063
emm12.0	**F7953**	<0.125	0.5	<0.125	1	1	0.031	0.063
emm4.0	**F7955**	<0.125	≥ 2(R)	<0.125	≥ 16(R)	0.5	0.031	≥ 1(R)
emm11.18	**F7956**	<0.125	≥ 2(R)	≥ 8(R)	1	2	≥ 1(R)	≥ 1(R)
emm81.0	**F7995**	<0.125	0.5	<0.125	1	0.5	0.063	0.063
emm12.0	**F8019**	<0.125	0.5	<0.125	1	0.25	0.031	0.063
emm12.7	**F8035**	<0.125	≥ 2(R)	≥ 8(R)	2	0.125	≥ 1(R)	≥ 1(R)
emm3.95	**F8038**	<0.125	0.063	<0.125	1	0.5	0.016	0.031
emm89.0	**FS27**	<0.125	0.25	<0.125	4	0.25	0.016	0.063
emm89.0	**FS28**	<0.125	0.5	<0.125	2	0.5	0.063	0.063
emm89.0	**FS29**	<0.125	0.5	<0.125	1	0.5	0.063	0.063
emm89.0	**FS30**	<0.125	0.5	<0.125	2	0.5	0.063	0.063
emm89.0	**FS31**	<0.125	0.5	<0.125	1	0.25	0.031	0.063
emm4.0	**F8123**	<0.125	0.5	<0.125	1	0.25	0.031	0.031
emm4.0	**F8152**	<0.125	0.5	≥ 8(R)	1	0.25	0.063	0.031
emm4.0	**F8153**	<0.125	1(I)	<0.125	1	0.25	0.031	0.063
emm4.0	**F8154**	<0.125	1(I)	<0.125	2	0.5	0.031	0.5(I)
emm11.1	**F8155**	<0.125	0.5	<0.125	1	2	0.063	0.063
emm3.95	**F8170**	<0.125	0.25	<0.125	1	1	0.031	0.063
emm3.95	**F8189**	<0.125	0.25	<0.125	1	1	0.031	0.063
emm4.0	**F8190**	<0.125	≥ 2(R)	<0.125	1	0.25	0.031	≥ 1(R)
emm12.0	**F8191**	<0.125	0.5	<0.125	2	0.25	0.063	0.063
emm3.95	**F8192**	<0.125	0.5	<0.125	1	1	0.063	0.063
emm12.0	**F8193**	<0.125	0.5	<0.125	1	0.25	0.063	0.063
emm4.0	**F8215**	<0.125	1(I)	<0.125	1	0.5	0.016	≥ 1(R)
emm4.0	**F8216**	<0.125	0.25	<0.125	0.5	0.25	0.031	0.063
emm11.1	**F8217**	<0.125	0.5	<0.125	1	1	0.031	0.063
emm4.0	**F8223**	<0.125	0.5	≥ 8(R)	1	0.5	<0.016	0.063
emm4.0	**F8225**	<0.125	1(I)	<0.125	1	0.25	0.016	0.5(I)
emm11.1	**F8227**	<0.125	≤0.031	<0.125	1	1	0.063	0.063
emm12.0	**F8239**	<0.125	0.5	<0.125	2	0.25	0.063	0.063
emm89.0	**FS32**	<0.125	0.5	<0.125	1	0.5	0.031	0.063
emm4.0	**F8264**	<0.125	0.25	≥ 8(R)	1	0.25	0.063	0.031
emm4.0	**F8295**	<0.125	≥ 2(R)	<0.125	1	0.5	0.031	≥ 1(R)
emm4.0	**F8308**	<0.125	0.5	<0.125	1	1	0.031	0.063
emm89.0	**FS33**	<0.125	0.125	<0.125	1	0.25	0.031	0.063
emm4.0	**F8323**	<0.125	0.5	<0.125	1	1	0.063	0.063
emm4.0	**F8324**	<0.125	0.25	<0.125	1	1	0.063	0.063
emm4.0	**F8333**	<0.125	0.25	<0.125	1	1	0.031	0.063
emm89.0	**FS34**	<0.125	0.5	<0.125	1	0.25	0.063	0.125
emm4.0	**F8335**	<0.125	0.125	<0.125	1	0.125	0.016	0.063
emm89.0	**FS35**	<0.125	0.5	<0.125	0.5	0.25	0.031	0.063
emm89.0	**FS36**	<0.125	0.25	<0.125	1	0.125	0.016	0.125
emm89.0	**FS37**	<0.125	0.5	<0.125	1	1	0.063	0.063
emm89.0	**FS38**	<0.125	0.5	<0.125	1	1	0.063	0.063
emm89.0	**FS39**	<0.125	0.125	<0.125	1	0.125	0.016	0.031
emm89.0	**FS40**	<0.125	0.5	<0.125	1	0.25	0.063	0.125
emm89.0	**FS41**	<0.125	0.5	<0.125	1	0.25	0.031	0.125
emm89.0	**FS42**	<0.125	0.5	<0.125	1	0.5	0.063	0.125
emm89.0	**FS43**	<0.125	0.5	<0.125	1	0.5	0.063	0.125
emm89.0	**FS44**	<0.125	0.5	<0.125	1	0.25	0.031	0.125
emm89.0	**FS45**	<0.125	≥ 2(R)	<0.125	1	0.5	0.25	≥ 1(R)
emm89.0	**FS46**	<0.125	0.5	0.5	2	0.5	≥ 1(R)	0.125
emm89.0	**TK43**	<0.125	≥ 2(R)	<0.125	1	1	0.5(I)	≥ 1(R)
emm89.0	**TK44**	<0.125	0.5	<0.125	1	1	0.063	0.063
emm89.0	**TK45**	<0.125	1(I)	<0.125	1	0.25	≥ 1(R)	0.5(I)
emm89.0	**TK46**	<0.125	0.5	<0.125	1	0.25	0.031	0.063
emm89.0	**TK47**	<0.125	0.5	<0.125	1	0.25	0.031	0.125
emm89.0	**TK48**	<0.125	0.25	<0.125	1	0.25	0.063	0.125
emm89.0	**TK49**	<0.125	0.25	0.125	1	0.25	0.063	0.063
emm89.0	**TK50**	<0.125	0.063	<0.125	1	0.25	0.063	0.125
emm89.0	**TK51**	<0.125	≥ 2(R)	<0.125	1	<0.125	≥ 1(R)	≥ 1(R)
emm89.0	**TK52**	<0.125	0.5	<0.125	1	0.5	0.125	0.125
emm89.0	**TK53**	<0.125	0.25	<0.125	1	0.25	0.016	0.063
emm89.0	**TK54**	<0.125	0.5	<0.125	1	0.25	0.063	0.125
emm89.0	**TK55**	<0.125	0.5	0.125	1	1	0.25	0.125
emm89.0	**TK56**	<0.125	0.5	<0.125	1	0.25	≥ 1(R)	0.125
emm89.0	**TK57**	<0.125	0.5	0.125	1	0.25	≥ 1(R)	0.125
emm89.28	**TK58**	<0.125	0.25	0.125	1	0.5	0.008	0.125
emm89.0	**TK59**	<0.125	0.5	<0.125	1	0.25	0.008	0.063
emm89.0	**TK60**	<0.125	≥ 2(R)	0.125	1	0.25	0.063	≥ 1(R)
emm89.0	**TK61**	<0.125	≥ 2(R)	0.125	2	0.125	≥ 1(R)	≥ 1(R)
emm89.0	**TK62**	<0.125	0.25	<0.125	1	0.25	0.063	0.063
emm89.0	**TK63**	<0.125	0.5	<0.125	1	0.25	≥ 1(R)	0.063
emm89.0	**TK64**	<0.125	0.125	<0.125	0.5	0.25	0.016	0.063
emm89.0	**TK65**	<0.125	0.063	<0.125	1	1	≥ 1(R)	0.063
emm89.0	**TK66**	<0.125	0.5	<0.125	0.5	0.25	0.063	0.063
emm89.0	**TK67**	<0.125	0.5	<0.125	1	0.5	0.031	0.063
emm89.0	**TK68**	<0.125	0.5	0.125	1	0.25	0.031	0.063
emm89.0	**TK69**	<0.125	≥ 2(R)	≥ 8(R)	1	0.25	0.063	≥ 1(R)
emm89.0	**TK70**	<0.125	0.25	<0.125	1	0.25	0.063	0.063
emm89.0	**TK71**	<0.125	≥ 2(R)	<0.125	1	0.125	≥ 1(R)	≥ 1(R)
emm89.0	**TK72**	<0.125	≥ 2(R)	<0.125	1	0.125	≥ 1(R)	≥ 1(R)
emm89.0	**TK73**	<0.125	0.5	<0.125	1	0.25	0.063	0.063
emm89.0	**TK74**	<0.125	≥ 2(R)	<0.125	1	0.5	≥ 1(R)	≥ 1(R)
emm89.0	**TK75**	<0.125	0.25	0.125	0.5	0.25	0.063	0.063
emm89.0	**TK76**	<0.125	≥ 2(R)	0.125	1	0.25	≥ 1(R)	≥ 1(R)
emm89.0	**TK77**	<0.125	0.5	<0.125	1	0.25	0.063	0.031
emm89.0	**TK78**	<0.125	≥ 2(R)	<0.125	1	0.25	≥ 1(R)	≥ 1(R)
emm89.0	**TK79**	<0.125	1(I)	<0.125	1	0.5	0.063	0.063
emm89.0	**TK80**	<0.125	≥ 2(R)	<0.125	1	0.25	≥ 1(R)	≥ 1(R)
emm89.0	**TK81**	<0.125	≥ 2(R)	<0.125	2	0.25	0.063	≥ 1(R)
emm89.0	**TK82**	<0.125	≥ 2(R)	<0.125	2	0.5	≥ 1(R)	≥ 1(R)
emm89.0	**TK83**	<0.125	≥ 2(R)	<0.125	1	0.125	0.063	≥ 1(R)
emm89.0	**TK84**	<0.125	0.5	<0.125	2	0.25	0.016	0.125
emm89.0	**TK85**	<0.125	0.5	<0.125	1	0.125	0.016	0.125
emm89.0	**TK86**	<0.125	1(I)	<0.125	1	0.25	0.031	0.125
emm89.0	**TK87**	<0.125	0.5	<0.125	1	0.25	0.016	0.125
emm89.0	**TK88**	<0.125	0.25	<0.125	1	0.25	0.031	0.063
emm89.0	**TK89**	<0.125	0.125	<0.125	1	0.125	0.016	0.125
emm89.0	**TK90**	<0.125	0.5	<0.125	1	0.25	0.031	0.063
emm89.0	**TK91**	<0.125	0.5	<0.125	2	0.25	0.063	0.125
emm89.0	**TK92**	<0.125	0.5	<0.125	0.5	0.25	0.016	0.125
emm89.0	**TK93**	<0.125	0.5	<0.125	1	0.25	0.016	0.125
emm89.0	**TK94**	<0.125	0.5	<0.125	1	0.5	0.031	0.063
emm89.0	**TK95**	<0.125	≥ 2(R)	4(I)	1	0.25	≥ 1(R)	≥ 1(R)
emm89.0	**TK96**	<0.125	≥ 2(R)	<0.125	1	0.25	≥ 1(R)	≥ 1(R)
emm89.0	**TK97**	<0.125	0.5	<0.125	1	0.25	0.031	0.063
emm89.0	**TK98**	<0.125	≥ 2(R)	<0.125	1	0.25	≥ 1(R)	≥ 1(R)
emm89.0	**TK99**	<0.125	≥ 2(R)	<0.125	1	0.25	≥ 1(R)	≥ 1(R)
emm89.0	**TK100**	<0.125	≥ 2(R)	<0.125	1	0.25	0.5(I)	≥ 1(R)
emm89.0	**TK101**	<0.125	0.5	<0.125	1	0.25	0.063	0.063
emm89.0	**TK102**	<0.125	≥ 2(R)	<0.125	1	0.25	≥ 1(R)	≥ 1(R)
emm89.0	**TK103**	<0.125	0.125	<0.125	1	0.125	0.031	0.125
emm89.0	**TK104**	<0.125	0.5	0.125	1	0.25	0.031	0.125
emm89.0	**TK105**	<0.125	0.5	<0.125	1	0.25	0.063	0.125
emm89.0	**TK106**	<0.125	0.25	<0.125	1	0.25	0.063	0.063
emm89.0	**TK107**	<0.125	≥ 2(R)	<0.125	1	0.125	0.063	≥ 1(R)
emm89.0	**TK108**	<0.125	≥ 2(R)	<0.125	1	0.25	≥ 1(R)	≥ 1(R)
emm89.0	**TK109**	<0.125	≥ 2(R)	<0.125	1	0.25	0.125	≥ 1(R)
emm89.0	**TK110**	<0.125	0.125	<0.125	1	0.125	0.016	0.063
emm89.0	**TK111**	<0.125	≥ 2(R)	<0.125	1	0.25	0.063	≥ 1(R)
emm89.0	**TK112**	<0.125	≥ 2(R)	<0.125	1	0.5	0.25	≥ 1(R)
emm89.0	**TK113**	<0.125	≥ 2(R)	<0.125	1	0.25	≥ 1(R)	≥ 1(R)
emm89.0	**TK114**	<0.125	≥ 2(R)	<0.125	2	0.25	≥ 1(R)	≥ 1(R)
emm89.0	**TK115**	<0.125	0.5	0.125	1	0.25	<0.016	0.125
emm89.0	**TK116**	<0.125	0.5	0.125	1	0.25	<0.016	0.063
emm89.0	**TK117**	<0.125	0.5	0.125	1	0.25	0.063	0.063
emm89.0	**TK118**	<0.125	≥ 2(R)	0.125	1	0.25	≥ 1(R)	≥ 1(R)
emm89.0	**TK119**	<0.125	0.25	<0.125	0.5	0.25	0.063	0.063
emm89.0	**TK120**	<0.125	≥ 2(R)	0.125	1	0.25	≥ 1(R)	≥ 1(R)
emm89.0	**TK121**	<0.125	0.25	0.125	1	0.5	0.016	0.063
emm89.0	**TK122**	<0.125	≥ 2(R)	<0.125	1	0.25	≥ 1(R)	≥ 1(R)
emm89.0	**TK123**	<0.125	0.5	0.125	1	0.25	0.063	0.125
emm89.0	**TK124**	<0.125	0.5	0.125	1	0.25	0.031	0.125
emm89.0	**YG01**	<0.125	0.5	0.125	1	0.25	0.031	0.125
emm89.0	**YG02**	<0.125	0.5	<0.125	1	0.25	0.063	0.25
emm89.0	**YG03**	<0.125	0.125	0.125	1	0.125	0.031	0.125
emm89.0	**YG04**	<0.125	0.5	0.125	1	0.25	0.031	0.125
emm89.0	**YG05**	<0.125	0.5	<0.125	2	0.25	0.031	0.125
emm89.0	**YG06**	<0.125	0.5	<0.125	1	0.25	0.016	0.063
emm89.0	**YG07**	<0.125	0.5	<0.125	1	0.25	0.031	0.063
emm89.0	**YG08**	<0.125	0.5	<0.125	1	0.5	0.063	0.125
emm89.0	**YG09**	<0.125	0.5	<0.125	1	0.25	0.063	0.125
emm89.0	**YG10**	<0.125	0.5	<0.125	1	0.5	0.063	0.125
emm89.0	**YG11**	<0.125	0.5	<0.125	1	0.25	0.063	0.125
emm89.0	**YG12**	<0.125	0.125	<0.125	1	0.25	0.031	0.125
emm89.0	**YG13**	<0.125	0.5	0.125	1	0.25	0.063	0.125
emm89.0	**YG14**	<0.125	0.5	<0.125	1	0.25	0.063	0.125
emm89.0	**YG15**	<0.125	0.5	0.125	1	0.25	0.063	0.125
emm89.0	**YG16**	<0.125	0.5	<0.125	1	0.25	0.063	0.125
emm89.0	**YG17**	<0.125	0.5	<0.125	1	0.25	0.063	0.063
emm89.0	**YG18**	<0.125	0.5	<0.125	1	0.5	0.063	0.125
emm89.0	**YG19**	<0.125	0.5	<0.125	1	0.25	0.063	0.125
emm89.0	**YG20**	<0.125	0.25	<0.125	1	0.125	0.031	0.125
emm89.0	**YG21**	<0.125	0.5	<0.125	1	0.25	0.063	0.125
emm89.0	**YG22**	<0.125	0.5	<0.125	1	0.5	0.063	0.125
emm89.0	**YG23**	<0.125	0.5	0.125	1	0.5	0.063	0.125
emm89.0	**YG24**	<0.125	0.5	<0.125	1	0.25	0.063	0.125
emm89.0	**YG25**	<0.125	0.5	<0.125	1	0.25	0.063	0.125
emm89.0	**YG26**	<0.125	0.5	0.125	1	0.5	0.063	0.125
emm89.0	**YG27**	<0.125	0.5	<0.125	1	0.5	0.063	0.125
emm89.0	**YG28**	<0.125	0.5	0.125	1	0.5	0.063	0.125
emm89.0	**YG29**	<0.125	0.25	<0.125	1	0.125	0.016	0.125
emm89.0	**YG30**	<0.125	0.5	<0.125	1	0.25	0.063	0.125
emm89.0	**YG31**	<0.125	0.5	<0.125	1	0.25	0.063	0.031
emm89.0	**YG32**	<0.125	0.5	0.125	1	0.5	0.063	0.063
emm89.0	**YG33**	<0.125	0.5	0.125	1	0.5	0.063	0.063
emm89.0	**YG34**	<0.125	0.5	<0.125	1	0.5	0.063	0.063
emm89.0	**YG35**	<0.125	0.5	0.125	1	0.5	0.063	0.063
emm89.0	**YG36**	<0.125	0.5	0.125	1	0.5	0.063	0.125
emm89.0	**YG37**	<0.125	0.25	0.125	1	0.25	0.016	0.125
emm89.0	**YG38**	<0.125	0.125	<0.125	1	0.25	0.016	0.125
emm89.0	**YG39**	<0.125	0.063	0.125	1	1	0.063	0.125
emm89.0	**OS01**	<0.125	0.063	0.125	0.5	0.125	0.016	0.031
emm89.0	**OS02**	<0.125	0.5	<0.125	1	0.25	0.031	0.125
emm89.0	**OS03**	<0.125	0.5	<0.125	1	0.25	0.031	0.125
emm89.0	**OS04**	<0.125	0.25	<0.125	1	1	0.031	0.25
emm89.0	**OS05**	<0.125	0.125	<0.125	1	0.25	0.016	0.125
emm89.0	**OS06**	<0.125	0.25	<0.125	0.5	0.5	0.063	0.031
emm89.0	**OS07**	<0.125	0.125	0.125	0.5	0.5	0.063	0.063
emm89.0	**OS08**	<0.125	0.5	0.25	1	1	0.063	0.25
emm89.0	**OS09**	<0.125	0.25	<0.125	1	0.25	0.031	0.25
emm89.0	**OS10**	<0.125	0.25	<0.125	0.5	0.125	0.016	0.125
emm89.0	**OS11**	<0.125	0.25	0.125	1	0.25	0.031	0.125
emm89.0	**OS12**	<0.125	0.5	0.125	1	0.25	0.031	0.031
emm89.0	**OS13**	<0.125	0.25	<0.125	0.5	0.25	0.031	0.063
emm89.0	**OS14**	<0.125	0.5	<0.125	0.5	0.25	0.063	0.125
emm89.0	**OS15**	<0.125	0.25	<0.125	1	0.5	0.063	0.063
emm89.0	**OS16**	<0.125	0.063	<0.125	0.5	0.25	0.031	0.063
emm89.0	**OS17**	<0.125	0.5	<0.125	1	0.5	0.063	0.063
emm89.0	**OS18**	<0.125	0.5	0.125	1	0.5	0.063	0.125
emm89.0	**OS19**	<0.125	≥ 2(R)	<0.125	2	0.5	≥ 1(R)	≥ 1(R)
emm89.0	**OS20**	<0.125	0.5	<0.125	1	0.5	0.063	0.125
emm89.0	**OS21**	<0.125	0.25	<0.125	1	0.25	0.031	0.063
emm89.0	**OS22**	<0.125	0.5	<0.125	1	1	0.063	0.125
emm89.0	**OS23**	<0.125	0.25	<0.125	1	0.5	0.031	0.063
emm89.0	**OS24**	<0.125	0.5	<0.125	1	0.25	0.031	0.125
emm89.0	**OS25**	<0.125	0.5	<0.125	2	1	0.031	0.125
emm89.0	**OS26**	<0.125	0.5	0.125	2	≥ 8(R)	0.063	0.125
emm89.0	**OS27**	<0.125	0.5	<0.125	2	≥ 8(R)	0.031	0.125
emm89.0	**OS28**	<0.125	0.063	<0.125	2	0.5	0.031	0.016
emm89.0	**OS29**	<0.125	≥ 2(R)	<0.125	2	0.25	≥ 1(R)	≥ 1(R)
emm89.0	**OS30**	<0.125	0.063	<0.125	1	0.25	<0.016	0.063
emm89.0	**OS31**	<0.125	0.5	<0.125	2	1	0.063	0.125
emm89.0	**OS32**	<0.125	0.5	<0.125	2	1	0.031	0.125
emm89.0	**OS33**	<0.125	0.25	<0.125	1	0.25	0.031	0.063
emm89.0	**OS34**	<0.125	0.5	<0.125	1	0.25	0.031	0.125
emm89.0	**OS35**	<0.125	0.5	0.125	2	0.25	0.031	0.125
emm89.0	**OS36**	<0.125	0.25	<0.125	1	1	0.031	0.063
emm89.0	**OS37**	<0.125	0.5	<0.125	1	1	0.063	0.125
emm89.0	**OS38**	<0.125	0.5	<0.125	2	0.25	0.031	0.125
emm89.0	**OS39**	<0.125	0.125	<0.125	1	0.25	0.016	0.125
emm89.0	**OS40**	<0.125	0.25	<0.125	1	0.25	0.031	0.125
emm89.0	**OS41**	<0.125	0.5	<0.125	2	0.5	0.031	0.25
emm89.0	**OS42**	<0.125	0.5	0.125	2	0.25	0.063	0.25
emm89.0	**OS43**	<0.125	0.5	<0.125	1	0.25	0.031	0.125
emm89.0	**OS44**	<0.125	0.25	<0.125	1	0.25	0.016	0.125
emm89.0	**OS45**	<0.125	0.5	0.125	2	0.25	0.031	0.125
emm89.0	**OS46**	<0.125	≥ 2(R)	<0.125	2	0.125	≥ 1(R)	≥ 1(R)
emm89.0	**OS47**	<0.125	0.5	<0.125	2	0.25	0.031	0.125
emm89.0	**OS48**	<0.125	0.25	<0.125	0.5	0.5	<0.016	0.031
emm89.0	**OS49**	<0.125	0.5	<0.125	1	1	0.063	0.125
emm89.0	**TK01**	<0.125	0.25	<0.125	2	0.25	0.031	0.125
emm89.0	**TK02**	<0.125	0.5	0.125	1	0.5	0.063	0.125
emm89.0	**TK03**	<0.125	0.5	0.125	1	0.5	0.031	0.125
emm89.0	**TK04**	<0.125	0.125	0.125	1	0.25	0.016	0.125
emm89.0	**TK05**	<0.125	0.5	0.125	2	0.5	0.063	0.125
emm89.0	**TK06**	<0.125	0.5	0.125	2	0.25	0.125	0.125
emm89.0	**TK07**	<0.125	0.5	<0.125	1	0.25	0.031	0.125
emm89.0	**TK08**	<0.125	≥ 2(R)	≥ 8(R)	2	0.25	≥ 1(R)	≥ 1(R)
emm89.0	**TK09**	<0.125	0.5	<0.125	1	0.125	<0.016	0.063
emm89.0	**TK10**	<0.125	0.5	0.25	2	1	0.063	0.125
emm89.0	**TK11**	<0.125	0.5	0.125	2	0.5	0.063	0.125
emm89.0	**TK12**	<0.125	0.5	0.125	2	1	0.063	0.125
emm89.0	**TK13**	<0.125	0.5	0.125	2	1	0.063	0.125
emm89.0	**TK14**	<0.125	0.5	0.125	1	1	0.063	0.063
emm89.0	**TK15**	<0.125	0.5	<0.125	2	0.5	0.063	0.063
emm89.0	**TK16**	<0.125	0.5	0.125	1	0.5	0.063	0.125
emm89.0	**TK17**	<0.125	0.25	0.125	2	0.125	0.016	0.125
emm89.0	**TK18**	<0.125	0.25	<0.125	1	0.5	0.031	0.063
emm89.0	**TK19**	<0.125	0.25	<0.125	1	0.25	0.031	0.063
emm89.0	**TK20**	<0.125	0.5	<0.125	1	0.25	0.063	0.125
emm89.0	**TK21**	<0.125	0.25	<0.125	1	0.5	0.031	0.25
emm89.0	**TK22**	<0.125	0.25	<0.125	1	0.25	0.063	0.25
emm89.0	**TK23**	<0.125	0.125	<0.125	1	0.25	0.031	0.25
emm89.0	**TK24**	<0.125	0.5	<0.125	1	1	0.063	0.125
emm89.0	**TK25**	<0.125	0.25	<0.125	1	0.125	0.031	0.125
emm89.0	**TK26**	<0.125	0.25	<0.125	1	0.25	0.063	0.125
emm89.0	**TK27**	<0.125	0.5	<0.125	1	0.25	0.063	0.125
emm89.0	**TK28**	<0.125	0.25	<0.125	1	0.25	0.063	0.125
emm89.0	**TK29**	<0.125	0.5	<0.125	1	1	0.063	0.125
emm89.0	**TK30**	<0.125	0.25	<0.125	1	0.5	0.031	0.125
emm89.0	**TK31**	<0.125	0.063	<0.125	1	0.25	0.016	0.125
emm89.0	**TK32**	<0.125	0.063	0.25	1	0.25	0.031	0.125
emm89.0	**TK33**	<0.125	0.25	<0.125	1	0.25	0.031	0.125
emm89.0	**TK34**	<0.125	0.5	<0.125	2	0.5	0.063	0.25
emm89.0	**TK35**	<0.125	0.5	<0.125	1	0.5	0.063	0.25
emm89.0	**TK36**	<0.125	0.5	<0.125	1	1	0.063	0.125
emm89.0	**TK37**	<0.125	0.5	<0.125	1	≥ 8(R)	0.063	0.125
emm89.0	**TK38**	<0.125	0.25	<0.125	1	0.5	0.063	0.125
emm89.0	**TK39**	<0.125	0.063	<0.125	1	0.25	0.016	0.125
emm89.0	**TK40**	<0.125	0.5	<0.125	1	0.5	0.031	0.125
emm89.0	**TK41**	<0.125	0.125	<0.125	1	0.25	0.031	0.125
emm89.0	**TK42**	<0.125	0.5	<0.125	1	0.5	0.063	0.125
emm89.0	**FS01**	<0.125	0.25	<0.125	1	0.25	0.031	0.125
emm89.0	**FS02**	<0.125	≥ 2(R)	<0.125	2	0.5	0.25	≥ 1(R)
emm89.0	**FS03**	<0.125	0.25	<0.125	1	0.25	0.031	0.063
emm89.0	**FS04**	<0.125	0.25	<0.125	2	0.25	0.031	0.063
emm89.0	**FS05**	<0.125	≥ 2(R)	<0.125	2	0.25	≥ 1(R)	≥ 1(R)
emm89.0	**FS06**	<0.125	0.125	<0.125	1	1	0.016	0.125
emm89.0	**FS07**	<0.125	0.25	<0.125	1	1	0.031	0.125
emm89.0	**FS08**	<0.125	0.25	<0.125	1	1	0.031	0.125
emm89.0	**FS09**	<0.125	0.5	<0.125	2	0.25	0.031	0.125
emm89.0	**FS10**	<0.125	0.5	<0.125	1	0.5	0.063	0.125
emm89.0	**FS11**	<0.125	0.125	<0.125	1	0.25	<0.016	0.063
emm89.0	**FS12**	<0.125	0.5	<0.125	1	0.25	0.031	0.063
emm89.0	**FS13**	<0.125	0.125	<0.125	1	0.5	0.031	0.125
emm89.0	**TY01**	<0.125	0.25	<0.125	1	0.25	0.031	0.25
emm89.0	**TY02**	<0.125	0.125	<0.125	1	0.25	0.016	0.063
emm89.0	**TY03**	<0.125	0.25	<0.125	1	0.25	0.016	0.125
emm89.0	**TY04**	<0.125	0.5	<0.125	1	1	0.063	0.125
emm89.0	**TY05**	<0.125	0.5	<0.125	1	1	0.031	0.063
emm89.0	**YH01**	<0.125	0.25	<0.125	1	1	0.031	0.125
emm89.0	**YH02**	<0.125	0.5	<0.125	1	1	0.063	0.125
emm89.0	**YH03**	<0.125	0.5	<0.125	1	0.25	0.063	0.125
emm89.0	**YH04**	<0.125	0.5	<0.125	1	0.25	0.063	0.125
emm89.0	**YH05**	<0.125	0.5	<0.125	1	0.25	0.063	0.125
emm89.0	**YH06**	<0.125	0.5	<0.125	2	0.25	0.063	0.125
emm89.0	**YH07**	<0.125	0.25	<0.125	1	0.25	0.031	0.125
emm89.0	**YH08**	<0.125	≥ 2(R)	<0.125	2	0.5	≥ 1(R)	≥ 1(R)
emm89.0	**YH09**	<0.125	0.125	<0.125	1	0.25	0.031	0.125
emm89.0	**YH10**	<0.125	0.5	<0.125	2	1	0.063	0.125
emm89.0	**YH11**	<0.125	0.25	<0.125	1	1	0.031	0.125
emm89.0	**YH12**	<0.125	0.5	<0.125	1	1	0.063	0.125
emm89.0	**YH13**	<0.125	0.25	<0.125	1	1	0.031	0.125

The detailed information of 368 *S. pyogenes* (including *emm* types) and their MIC to seven antibiotics (PG, ERY, AZM, TC, LFX, CHL and CLI) is presented in the table.

**Figure 2.**
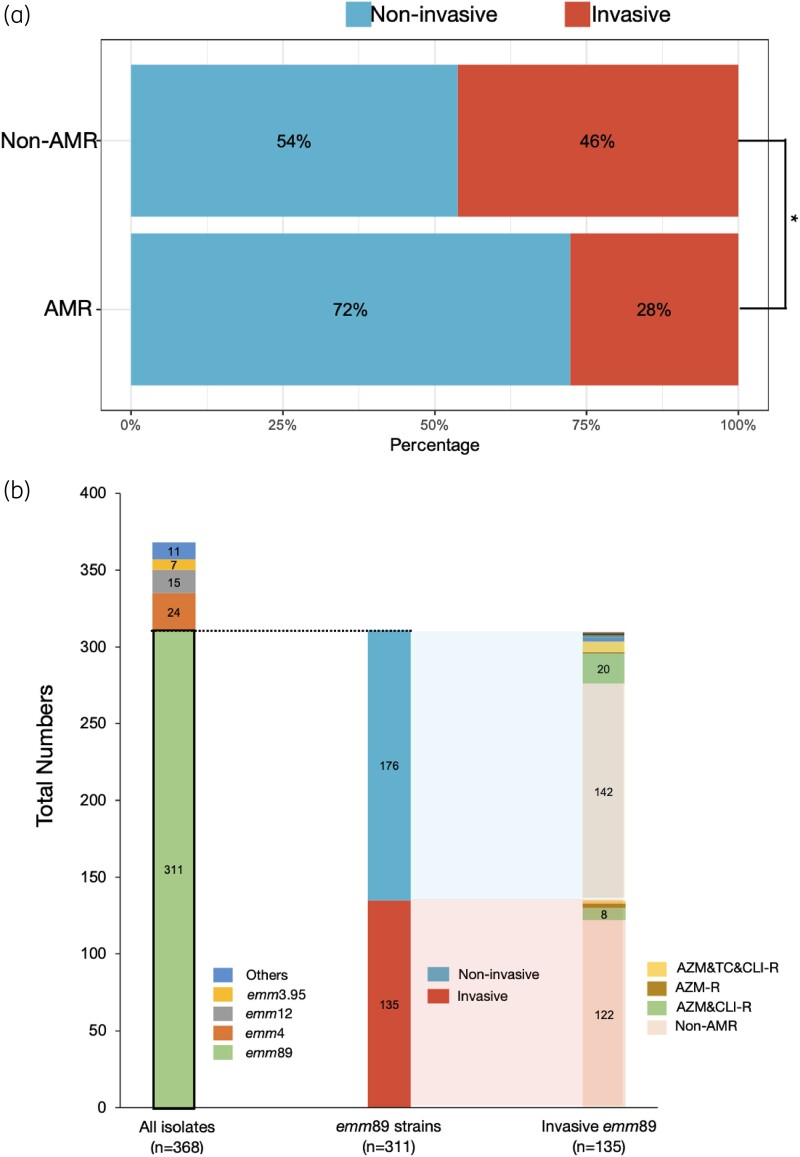
AMR occurs more frequently in non-invasive *S. pyogenes* infection. (a) Percentage stacked bar chart showing the percentage of AMR and non-AMR *S. pyogenes emm*89 causing invasive and non-invasive infections. The data indicate a significant correlation of non-invasive infections to AMR as compared with invasive infections. *, *P* < 0.05; χ^2^ test, *P *= 0.01806; n_AMR _= 47; n_non-AMR _= 264. (b) Stacked bar chart showing the distribution of *emm*89 strains in all 368 strains and the number of host patients with invasive or non-invasive infections. AMR status in invasive strains was analysed further.

Whereas, AMR narrows the options for clinical treatment, most AMR mechanisms are associated with a fitness cost that is typically observed as a reduced bacterial growth rate.^17^ We studied the correlation between AMR and clinical diagnoses and statistically analysed the occurrence of AMR in *emm*89 isolates from patients with either invasive or non-invasive infections. As a result, we found that the proportion of invasive *S. pyogenes* infection-derived strains showing AMR was 28%, which was lower than that of invasive *S. pyogenes* infection-derived strains sensitive to all antibiotics (46%, χ^2^ test, *P *= 0.018; Figure 2a and b). Therefore, we concluded that there was a negative correlation between AMR and invasiveness in *emm*89 *S. pyogenes* isolates from Japan.

We investigated the relationships between AMR and genotypes, including clades in *emm*89 and multi-locus sequence typing (MLST). ST101, a dominant phylogeny in *emm*89 strains, was strongly related to levofloxacin-resistant strains (*P *< 0.001; pairwise χ^2^ test with Bonferroni’s correction). No significant correlation between other MLSTs or clades with AMR was observed.

### Identification of AMR-associated genes in S. pyogenes using genomic analysis

**Figure 3.**
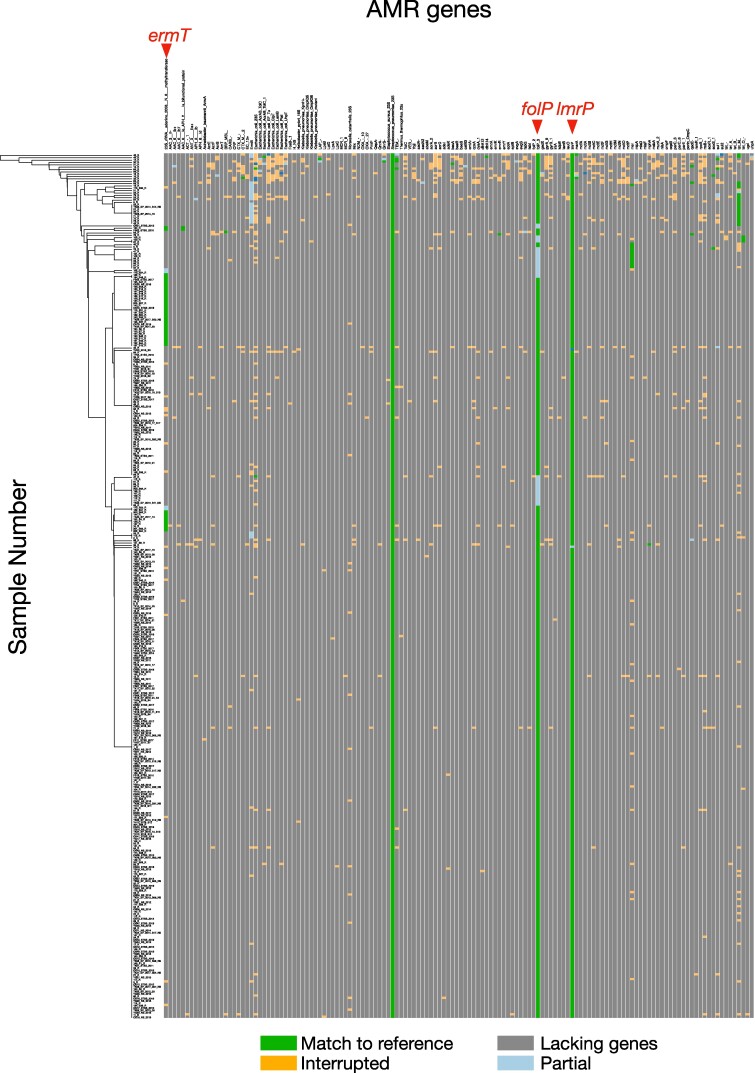
Burden of AMR genes in the clinical strains including *S. pyogenes.* The reference data were obtained from the CARD. Green, light blue, orange and grey indicate matches to reference, interrupted, partial and lacking genes, respectively. ‘Interrupted’ refers to an incomplete gene, lacking start and/or stop codons; ‘fragmented’ refers to a gene assembled to ≥2 contigs; ‘partial’ indicates that not all of the reference is represented in the assembly. The distribution of AMR genes in each strain is also shown in Table S2. The clustering tree was generated using ARIBA based on the gene distribution. Graphical data were obtained using Phandango. The 23S rRNA gene, *ermT* (23S_rRNA_adenine_2058_N_6_methyltransferase), *folP, ImrP* and *tetM* were determined to contain known variants contributing to AMR.

In a previous study, Ikebe *et al.* found that *ermA*, *ermB* and *mefA/E* were abundant in *emm*1 strains resistant to ERY and/or clindamycin but barely existed in *emm*89.^3^ To detect the genes contributing to the exhibited AMR patterns, the presence of reported AMR-associated genes in the whole genome of *emm*89 *S. pyogenes* was examined by sequence-based profiling using ARIBA and CARD (Figure 3). The *ermT* gene, a 23S ribosomal RNA methyltransferase, was detected in 42 strains, all of which were *emm*89 (Figure 3; Table S2). Although we did not find *emm*89 strains carrying full-length *ermB* using sequencing-based profiling in CARD, a BLAST search confirmed the presence of complete *ermB* sequences in the genomes of seven isolates. Of the isolates, five displayed AZM&CLI&TC patterns, whereas one displayed AZM&CLI patterns. The other strains showed intermediate susceptibility to AZM (Table S4). Neither *ermB* nor *ermT* were detected in eight strains belonging to AZM&CLI&TC or AZM&CLI; hence, sequence-based profiling was performed using MEGARes and the AMR++ pipeline, indicating that *ermB*, *ermA* and *mefA* were detected in five, two and one strains, respectively (Table S5).

The gene, *tetM*, was shared among all TC-resistant strains. Three levofloxacin-resistant strains were found; however, we were unable to detect any known quinolone resistance genes associated with levofloxacin resistance even using ARIBA, MEGARes and AMR++. This suggests that the three resistant strains may have acquired novel gene(s) or mutation(s) conferring quinolone resistance. However, *folP* and *lmrP* were found in almost all *emm*89 and other *emm*-type strains. The *folP* gene has been shown to confer sulfonamide resistance and *lmrP* encodes a putative multidrug resistance efflux; however, the natural substrate of LmrP in *S. pyogenes* is unknown.^18–20^ Additionally, only in *emm*4 strains, *tetO* was detected in TC-resistance and *soxS* and *mel* was detected in chloramphenicol-resistance (Figure 3; Table S2). Taken together, all observed resistances, except for levofloxacin, were explained by the presence of known AMR-related genes as determined using sequence-based profiling.

### Detection of SNPs/indels and genes associated with AMR using GWAS

**Figure 4.**
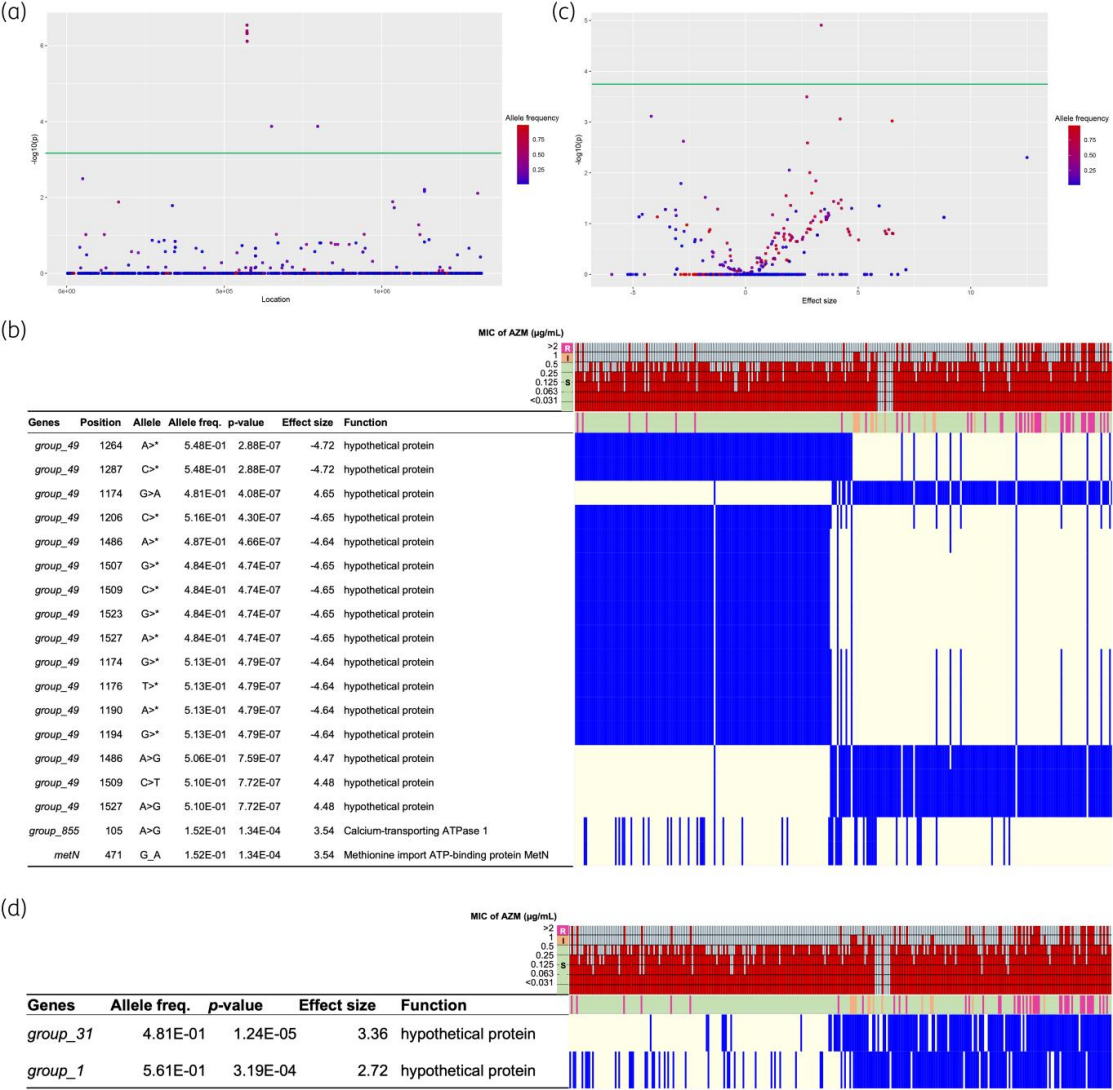
GWAS regarding ERY resistance. (a) Manhattan plot—the X-axis shows the location of each SNP/indel on the core gene alignment, whereas the Y-axis indicates the *P* value. The significance level was set as *P *= 6.56 × 10^−4^ using a permutation test and is shown as a green line. Plots are coloured based on the allele frequency. (b) Distribution heatmap for the variants strongly related to resistance, with the strains possessing the significant SNPs/indels coloured. The colour bars and bar plot indicate the MIC of each strain. Resistant (R; in magenta), intermediate (I; in yellow) and susceptible (S; in green) are determined according to the MIC > 2, =1, and <1, respectively. The position is the location of each variant within the respective gene locus. (c) Volcano plot—the X-axis shows the effect size, whereas the Y-axis indicates the *P* value. The significance level was set as *P *= 1.77 × 10^−4^ using a permutation test and is shown as a green line. (d) Distribution heatmap for genes, with the strains possessing the significant genes coloured. SNP, single-nucleotide polymorphism; indel, insertion/deletion; MIC, minimal inhibitory concentration.

We also attempted to identify new AMR-associated variants and genes in these samples. GWASs were performed targeting SNPs/indels in core genes harboured in more than 99% of all isolates and in the presence or absence of all genes. Analysis of SNPs/indels indicated two SNPs that were strongly related to AZM resistance (Figure 4a and b). Significant SNPs were found in the *group_855* and *metN* loci, which encode calcium-transporting ATPase (MGAS27061_0531) and methionine-importing ATP-binding protein, respectively. However, both SNPs caused synonymous amino acid substitutions. Using GWAS to target the presence of genes, one gene that was strongly associated with AZM resistance, *group_31*, which encodes collagen-like surface protein A (SclA), was detected (Figure 4c and d). The gene with the second-lowest *P* value was *group_1*, which also encoded the collagen-like protein, SclB, although the difference was not statistically significant. Taken together, these results suggest that novel genetic factors may assist in the development of AMR resistance in *S. pyogenes*.

## Discussion

In the present study, we measured the MIC of 311 *emm*89 *S. pyogenes* isolates from Japan and found that 43 *emm*89 isolates showed a high resistance rate to AZM and/or clindamycin. AMR-associated genes were investigated by referring to databases and gene abundance in different *emm* types was compared and the possible genes associated with AZM-, AZM&CLI- and AZM&CLI&TC-resistant *emm*89 isolates were identified. We investigated the resistance of *emm*89 *S. pyogenes* to two macrolides (AZM and ERY). Although ERY is more commonly used in the *S. pyogenes* MIC assay than AZM,^21,22^ AZM plays a crucial role in clinical therapy due to its distinct advantage of once-daily dosing.^23^ Additionally, the results indicated that 42 *emm*89 isolates were simultaneously resistant to AZM and ERY (Figure S2). Twelve isolates showed susceptibility to ERY but intermediate susceptibility to AZM (Figure S2; Table S3). Although the resistance of all 368 *emm*89 *S. pyogenes* isolates to seven antibiotics were measured according to the CLSI standard, the power of the GWAS was low due to the small number of resistant bacteria, and only SNPs and genes correlated with AZM resistance were detected. More functional analysis is needed to prove the effect of genes/mutations on the resistant phenotypes.

A total of 13.8% (43/311) of *emm*89 *S. pyogenes* showed resistance to AZM and/or clindamycin. A study reported that *S. pyogenes* was resistant to macrolides and lincosamides, both in Japan and overseas.^24^ The existence of AZM and/or clindamycin-resistant *S. pyogenes* has recently posed a threat to public health in Japan by limiting drug options for *S. pyogenes* infection, as AZM and clindamycin still play a key role in treatment.^4^ In penicillin-allergic patients, macrolides, such as ERY and AZM, are the main alternatives. Regardless of the presence of penicillin allergy, if the disease progresses to an invasive infection, adjunctive clindamycin treatment can increase survival rates and improve the outcomes of patients with invasive *S. pyogenes* infections.^4^ This may be because clindamycin, a protein synthesis inhibitor, decreases the expression of bacterial virulence factors and exotoxins, although the underlying mechanism remains vague.^25^ Therefore, clindamycin plays an important role in the treatment of *S. pyogenes* infections, particularly invasive ones.

We performed high-throughput screening for AMR-related factors using sequence-based profiling and GWASs. Previous studies have detected specific AMR-related genes using polymerase chain reaction; the analysis in the current study provides a comprehensive detection of the AMR of *emm*89 *S. pyogenes* prevalent in Japan. A study tested the AMR of *emm*89 against ERY and clindamycin and detected the presence of *ermA*, *ermB* and *mefA/E* in their isolates, mainly using PCR.^3^ However, PCR can detect only predetermined targets. In the current study, the genome of all *emm*89 strains were sequenced, ensuring high-throughput screening for AMR-related factors using sequence-based profiling and GWASs. Using this approach, *ermT* was newly detected in AZM- and clindamycin-resistant *emm*89 strains.

Ikebe *et al.* characterized the *emm* types and antimicrobial susceptibility of isolates from patients with STSS in Japan.^3^ They found that 36% of STSS cases in 2018 were attributable to *emm*89 *S. pyogenes*, making it the second most prevalent type after *emm*1.^3^ They found that 10.34% (15/145) of clindamycin-resistant and 11.03% (16/145) of ERY-resistant *S. pyogenes* isolates from patients with STSS in Japan were *emm*89. These results are consistent with the current findings that 11 and 13 of 135 STSS-derived *emm*89 isolates were clindamycin- and AZM-resistant, respectively.^3^ However, the AMR-associated genes, *ermA, ermB* and *mefA/E,* which were most abundant in *emm*1 isolates, were never or rarely identified in *emm*89.^3^ In the current study, AZM and/or clindamycin-resistant isolates were the major populations in the AMR *emm*89 strains (43/47), and an abundance of *ermT* was observed in the *emm*89 strains (42/311). We assumed that the main determinant of resistance in *emm*89 strains was *ermT* because a previous study reported that *ermT* contributed to ERY resistance and inducible clindamycin resistance in the absence of *ermB* and *mefA/E* among *emm* 92, *emm*3, *emm* 9 and *emm*28 strains.^26^ To our knowledge, the current study is the first to report that *ermT* confers resistance to AZM and clindamycin in *emm*89 *S. pyogenes* in Japan. One strain harbouring *mefA/E* and *msrD*, which encodes the cytoplasmic ATPase counterpart of MefA/E, was detected. No previous reports describing the presence of *mefA/E* in *emm*89 strains isolated in Japan were found.

In the current study, a higher correlation between AMR and non-invasive infections than with invasive infections was found. The relationship between AMR and invasive *S. pyogenes* infection remains unclear. Scientists have found that hospital-acquired *Escherichia coli* and *Klebsiella pneumoniae* isolates obtained from patients with invasive bacterial infections are more resistant to multiple antibiotics than community-acquired isolates in Cambodia.^27^ In contrast, AMR in *S. pneumoniae* was detected more frequently in non-invasive than in invasive isolates from adults in Spain.^28^ The current results are consistent with those of the latter study. A possible explanation for this observation is that AMR-associated factors might be fitting for *S. pyogenes* and affect the efficient utilization of ATP for host invasion.

GWASs were performed to identify novel factors that confer resistance. AZM resistance-associated genetic factors were identified, including SNPs in calcium-transporting and methionine-transport ATPases and the presence of the *sclA* gene, encoding a collagen-like surface protein. As no studies have described the relationship between these factors and macrolide resistance, their effects on macrolide resistance might be relatively low. Several studies have reported that another surface protein, PrtF1/SfbI, a virulence factor involved in host cell invasion, is associated with ERY resistance.^29,30^

In addition to the AZM and/or clindamycin-resistant *emm*89 strains, three *emm*89 isolates showed resistance to levofloxacin, whereas all three isolates were susceptible to both AZM and clindamycin. Tatara *et al.* reported that 33 strains were fluoroquinolone-resistant, including *emm*6 (14 strains, 42.4%), *emm*89 (10 strains, 30.3%), *emm*75 (4 strains, 12.1%), *emm*1 (3 strains, 9.1%), *emm*12 (1 strain, 3.0%) and *emm*44 (1 strain, 3.0%).^31^ Although *emm*89 strains seem to be the predominant *emm* type in Japan, the AMR genes conferring levofloxacin resistance have not yet been investigated. We could not elucidate the AMR-related genes or mutations conferring levofloxacin resistance in *emm*89 strains in this study because of the small quantity of levofloxacin-resistant *emm*89 strains. In the current study, the common fluoroquinolone-resistance genes, like *gyrA* (DNA gyrase subunitA) and *parC,* were not detected in all three levofloxacin-resistant *emm*89 strains. One possible hypothesis is that levofloxacin resistance may be related to different alterations happening in gyrase-encoding genes or *parC*, which makes it difficult to detect the common gyrase-encoding genes or *parC* in the CARD database. However, no specific nucleic acid mutants or SNPs for *parC* or *gyrA* were reported in relation to levofloxacin resistance. GWASs were not feasible in this study because of the small quantity of levofloxacin-resistant *emm*89 strains. Lin *et al.* reported that amino acid alterations in Ser79Phe and Ala12Val in ParC were the most common mutations in fluoroquinolone-resistant *emm*12 *S. pyogenes* in Taiwan.^32^

In conclusion, 15.11% (47/311) of isolates were AMR *emm*89 *S. pyogenes* belonging to seven patterns (Figure 1a), whereas 91.49% (43/47) of these were resistant to AZM and/or clindamycin; additionally, seven AMR patterns were identified. As 89% of AMR genes were not identified in *emm*89 strains, we examined the reported AMR-associated genes using ARIBA and CARD and searched for new AMR-associated genes using GWASs. Consequently, *ermT*, *folP* and *lmrP* were found to exist abundantly in *emm*89 strains; *group_31*, encoding collagen-like surface protein A, was strongly associated with AZM resistance. The correlation between AMR *emm*89 and STSS indicates that non-invasive phenotypes are correlated with AMR. This study comprehensively analysed AMR *emm*89 collected throughout Japan from 2011 to 2021. The accumulation of bacterial genomic information along with MIC data are expected to improve the resolution of AMR gene distribution, increase the power of GWAS and elucidate currently unidentified factors.

## Supplementary Material

dlaf017_Supplementary_Data

## Data Availability

Data for the 207 sequenced *S. pyogenes* genomes were deposited in the DDBJ sequence read archive, under BioProject PRJDB16457. The DRR run number was DRR511668-DRR511874.

## Supplementary data

Figures S1 and S2 and Tables S1 to S5 are available as Supplementary data at *JAC-AMR* Online. Figures S1 and S2, and Tables S1 and S3 to S5 are provided as a PDF file. Table S2 is provided as an Excel file.
