# Supplementary material for: A comprehensive analysis of antimicrobial resistance of clinical emm89 Streptococcus pyogenes in Japan
Source: JAC Antimicrob Resist. 2025 Feb 19;7(1):dlaf017. doi: 10.1093/jacamr/dlaf017 (PMC11836879; doi:10.1093/jacamr/dlaf017)
Supplement: dlaf017_Supplementary_Data [file dlaf017_supplementary_data.zip › Supplementary Figures_R.pdf]

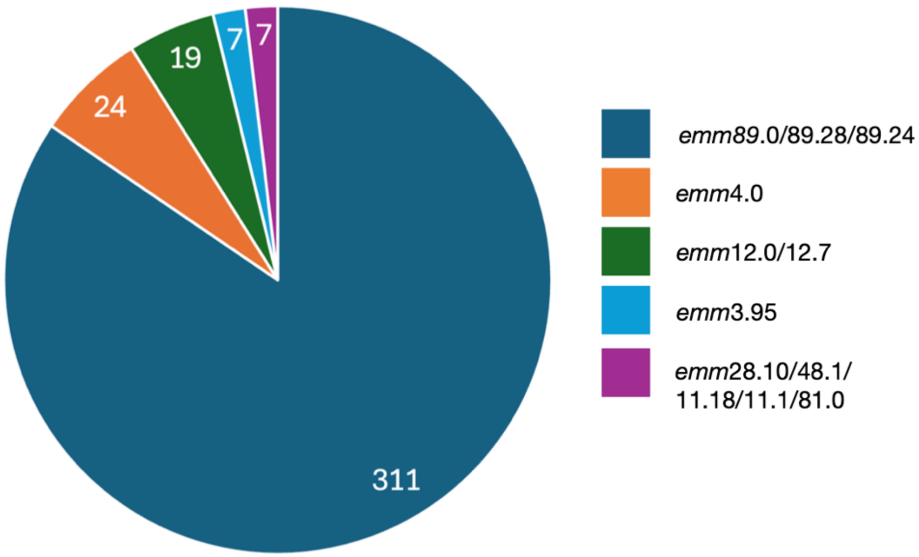

Figure S1. *S. pyogenes* information used in MIC assay.

Of the 368 isolated strains, 311 were *emm*89, 24 were *emm*4.0, 19 were *emm*12, and 7 were *emm*3.95. The remaining 7 strains were named *emm*28, *emm*48, *emm*11, *emm*81.

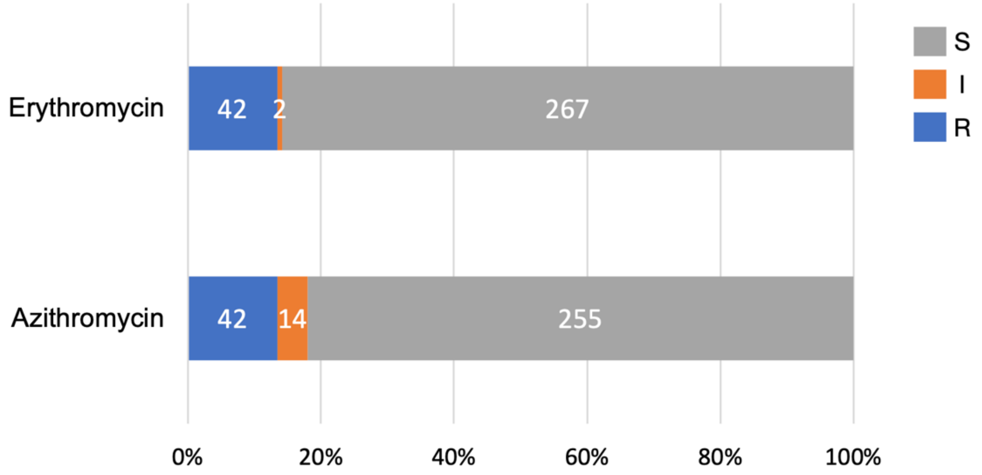

Figure S2. Comparison of *emm*89 *S. pyogenes* resistance to erythromycin and azithromycin.

Of all 311 *emm*89 isolates, 42 isolates were resistant to both erythromycin and azithromycin, and 12 isolates were sensitivity to erythromycin but intermediate susceptibility to azithromycin.

Table S1. Detailed information about antibiotics used in the study.
Detailed information regarding the antibiotics used for *S. pyogenes* MIC assay standards issued by the CLSI.

Name	Type	Target	Abbreviation	MIC assay Concentration(mg/L)
Penicillin-G	β-lactam antibiotics	Cell walls	PG	0.13~8
Azithromycin	Macrolide antibiotic	50S ribosome	AZM	0.031~2
Tetracycline	Tetracycline antibiotics	30S and 50S ribosome	TC	0.125~8
Chloramphenicol	Broad-spectrum antibiotic	50S ribosome	CHL	0.25~16
Levofloxacin	Fluoroquinolone	DNA synthesis	LFX	0.125~8
Clindamycin	Lincomycin antibiotics	50S ribosome	CLI	0.016~1
Erythromycin	β-lactam antibiotics	Cell walls	ERY	0.016~1

Table S3. Detailed information about 12 *emm*89 isolates that sensitive to erythromycin but intermediate to azithromycin.

Strain	Erythromycin	Azithromycin
KB01	Sensitive	Intermediate
KB02	Sensitive	Intermediate
KB03	Sensitive	Intermediate
KB04	Sensitive	Intermediate
KB11	Sensitive	Intermediate
KB12	Sensitive	Intermediate
YG40	Sensitive	Intermediate
FS20	Sensitive	Intermediate
FS26	Sensitive	Intermediate
TK45	Sensitive	Intermediate
TK79	Sensitive	Intermediate
TK86	Sensitive	Intermediate

Table S4. ***ermB*** **BLAST in bacterial genome determination.**

Only interrupted or partial *ermB* can be detected using ARIBA v.2.14.4, referring to the CARD. However, 13 *emm*89 isolates were confirmed to carry *ermB* by searching for *ermB* in BLAST.

**BLAST hit**	name	ErmB.assembled
	FS36	interrupted
	FS44	partial
	FS45	partial
	TK48	partial
	TK56	partial
	TK95	interrupted
	YG43	partial
	TK103	partial
	YG45	interrupted
	OS55	interrupted
	OS58	interrupted
	KB02	interrupted
	OS59	partial
	OS62	interrupted
	F7401	interrupted
	F7419	partial
	KB03	partial
**Yes**	F7469	interrupted
**Yes**	F7515	interrupted
**Yes**	F7517	interrupted
	F7529	interrupted
	F7544	partial
**Yes**	F7587	interrupted
**Yes**	F7601	interrupted
	KB05	partial
	FS23	partial
**Yes**	F7886	interrupted
**Yes**	F7920	interrupted
**Yes**	F7924	interrupted
**Yes**	F7936	interrupted
**Yes**	F7956	interrupted
**Yes**	F8035	interrupted
	F8038	partial
	FS30	partial
	F8192	partial
	F8217	partial
**Yes**	TK08	interrupted
**Yes**	TK33	interrupted

Table S5. Sequence-based profiling using MEGARes and AMR++.
Genes are reference sequences registered in MEGARes. The hits and gene fractions represent the number and coverage of short-read sequences aligned with the reference, respectively.

Strains	Genes	Hits	Gene Fraction
KB08	MEG_3806|Drugs|MLS|MLS_resistance_MFS_efflux_pumps|MEFA	2208	99.0156
	MEG_4073|Drugs|MLS|MLS_resistance_ABC_efflux_pumps|MSRD	8682	99.9317
OS55	MEG_2801|Drugs|MLS|23S_rRNA_methyltransferases|ERMB	1689	99.8689
OS58	MEG_2799|Drugs|MLS|23S_rRNA_methyltransferases|ERMB	894	96.9251
	MEG_2801|Drugs|MLS|23S_rRNA_methyltransferases|ERMB	638	99.8689
	MEG_2793|Drugs|MLS|23S_rRNA_methyltransferases|ERMB	618	99.8647
OS62	MEG_2801|Drugs|MLS|23S_rRNA_methyltransferases|ERMB	1439	99.8689
TK08	MEG_2801|Drugs|MLS|23S_rRNA_methyltransferases|ERMB	1202	99.8689
TK95	MEG_2801|Drugs|MLS|23S_rRNA_methyltransferases|ERMB	2622	99.7379
	MEG_2787|Drugs|MLS|23S_rRNA_methyltransferases|ERMA	13	95.6344
YH08	MEG_2787|Drugs|MLS|23S_rRNA_methyltransferases|ERMA	24	93.1787
